# The clinical, radiological and genetic spectrum *AARS2*-related leukoencephalopathy: a case series of 15 patients and review of the literature

**DOI:** 10.1007/s00415-026-13964-1

**Published:** 2026-06-26

**Authors:** Charles Wade, Anderson Rodrigues Brandão de Paiva, Paulo Ribeiro Nóbrega, Ana C Andorinho de Freitas Ferreira, Ana C Martins Borges, Lecio Figueira Pinto, Paula Brandão, Pedro Maia Nobre Rocha Saffi, Luiz Felipe Vasconcellos, Deborah Moreira Rangel, Paula Camila A. A. P. Matos, André Luiz Santos Pessoa, Emília K. Embiruçu, Katiane Sayão Souza Cabral, Fernando Kok, Rumana Chowdhury, Jessica Roberts, Jeremy Chataway, Frederik Barkhof, Tom Hayton, Matthew E. Adams, David S. Lynch

**Affiliations:** 1 Department of Neuroinflammation, UCL Queen Square Institute of Neurology, London, UK; 2 Hospital das Clínicas da Universidade de São Paulo, Neurogenetics Unit, São Paulo, Brazil; 3 Hospital Universitário Prof. Edgard Santos, Salvador, Brazil; 4 Department of Clinical Medicine, Universidade Federal Do Ceará, Fortaleza, Brazil; 5 Hospital Federal Dos Servidores Do Estado Do Rio de Janeiro, Rio de Janeiro, Brazil; 6 Faculdade de Medicina Do ABC, Santo André, Brazil; 7 Hospital Universitário de Brasília, Universidade de Brasília (HUB-UnB), Brasília, Brazil; 8 Instituto de Ensino E Pesquisa Do Hospital Sírio-Libanês (IEP-HSL), Brasília, Brazil; 9 Instituto de Neurologia Deolindo Couto, Federal University of Rio de Janeiro, Rio de Janeiro, Brazil; 10 Hospital Geral de Fortaleza, Fortaleza, Brazil; 11 Center of Health Sciences, Universidade Estadual Do Ceará, Fortaleza, Brazil; 12 Hospital Albert Sabin, Fortaleza, Brazil; 13 Child Neurology Division, Department of Neurology, Hospital das Clínicas da Faculdade de Medicina, Universidade de São Paulo (HC-FMUSP), São Paulo, Brazil; 14 Mendelics Genomic Analysis, São Paulo, Brazil; 15 Department of Neurology, Leeds Teaching Hospital NHS Trust, Leeds, UK; 16 National Institute for Health Research, University College London Hospitals Biomedical Research Centre, London, UK; 17 Lysholm Department of Neuroradiology, National Hospital for Neurology and Neurosurgery, London, UK; 18 Department of Radiology & Nuclear Medicine, Amsterdam UMC, Amsterdam, Netherlands; 19 Department of Neurology, University Hospitals Birmingham NHS Trust, Birmingham, UK

**Keywords:** AARS2, ALSP, Leukodystrophy, MRI

## Abstract

**Background:**

*AARS2*-related leukoencephalopathy is a rare autosomal recessive leukodystrophy with overlapping clinical and radiological features with CSF1R-related disease but distinct genetic and phenotypic characteristics. Since its description in 2014, fewer than 60 cases have been reported.

**Methods:**

We conducted a multicentre retrospective case series of individuals with genetically confirmed *AARS2*-related leukoencephalopathy, integrating clinical, radiological, and molecular data from 15 newly identified patients. Neuroimaging was reviewed by experienced neuroradiologists, and variants were classified using ACMG criteria. A systematic literature review identified 56 additional cases.

**Results:**

Across 71 individuals, mean age at onset was 27.3 years (range 0–57), with balanced sex distribution. Psychiatric and cognitive symptoms predominated early, affecting 70% and 86% of patients. Pyramidal signs (82%), gait disturbance (80%), and extrapyramidal motor features (45%) were common, whilst seizures occurred in 10%. Premature ovarian failure was reported in 82% of females. Sixteen individuals (22.5%) had childhood onset. Neuroimaging showed confluent frontoparietal white matter abnormalities, corpus callosum thinning, and multifocal diffusion abnormalities. Seventy-two unique *AARS2* variants were identified, including seven novel variants. Missense variants predominated. Notably, variants previously linked only to infantile cardiomyopathy, including p.Arg592Trp, were observed.

**Conclusions:**

*AARS2*-related leukoencephalopathy is clinically and genetically heterogeneous with characteristic imaging and broad onset. Recognition of defining features is essential for diagnosis, and genomic testing should be prioritised. This study provides the most comprehensive characterisation to date and suggests genotype–phenotype separation between cardiomyopathy- and leukodystrophy-associated variants may not be absolute.

**Supplementary Information:**

The online version contains supplementary material available at 10.1007/s00415-026-13964-1.

## Introduction

Leukodystrophies are a genetically and clinically heterogeneous group of inherited disorders characterised by progressive degeneration of central nervous system white matter due to abnormalities in myelin development, maintenance, or turnover. Traditionally regarded as paediatric-onset conditions, adult-onset leukodystrophies are now increasingly recognised, a shift driven by advances in neuroimaging and the widespread adoption of next-generation sequencing [1].

Amongst the expanding spectrum of monogenic causes, biallelic pathogenic variants in *AARS2*, which encodes the mitochondrial alanyl-tRNA synthetase (mt-AlaRS), have been established as a distinct cause of adult-onset leukodystrophy [2, 3]. *AARS2*-related leukoencephalopathy (MIM #615889) shares clinical and radiological features with Colony-Stimulating Factor 1 Receptor-related disorder (*CSF1R*-RD; MIM #221820) but is differentiated by its autosomal recessive inheritance pattern, earlier age of onset, and certain clinical and radiological features [4].

Since the first description in 2014, *AARS2*-related leukoencephalopathy has remained a rare diagnosis, with only 56 genetically confirmed cases reported across 31 publications [2, 5–34] These reports describe a recognisable clinical syndrome typically characterised by insidious psychiatric change and cognitive decline in early to mid-adulthood, followed by a progressive motor disorder incorporating pyramidal, cerebellar, and extrapyramidal signs. A childhood-onset syndrome with developmental delay can also occur.

Neuroimaging commonly shows confluent frontoparietal white matter abnormalities with relative U-fibre sparing, thinning of the corpus callosum, and distinctive abnormal foci on diffusion-weighted imaging (DWI).

Here, we present a multicentre case series of 15 individuals with genetically confirmed *AARS2*-related leukoencephalopathy and synthesise these findings with a literature review of all 56 published cases, providing the most comprehensive analysis of *AARS2*-related leukoencephalopathy to date.

We describe the clinical heterogeneity, radiological patterns, and genetic architecture of the disorder, explore genotype–phenotype relationships, and outline diagnostic principles relevant to both paediatric and adult-onset presentations.

## Methods

### Case ascertainment

We conducted a retrospective case series of individuals with genetically confirmed *AARS2*-related leukoencephalopathy, identified through clinical and research collaborations at multiple centres across the United Kingdom and Brazil. Cases were included if individuals presented with a leukoencephalopathy and had biallelic pathogenic or likely pathogenic variants in *AARS2*. Clinical, neuroimaging, and genetic data were collected from medical records using a standardised case reporting form. Contributing clinicians confirmed the diagnosis and provided longitudinal data where available.

### Ethical considerations

This study was approved by the ethics committee under UCL ION code 10/H0721/87. Informed consent was obtained for all living participants where required. This study was conducted in accordance with the principles of the Declaration of Helsinki.

### Genetic analysis

Molecular diagnoses were established through various platforms including targeted gene panels for leukodystrophy or mitochondrial disease, whole-exome sequencing, or Whole-genome sequencing, depending on the centre. Variant classification was based on American College of Medical Genetics and Genomics (ACMG) criteria, and compound heterozygosity was confirmed by segregation analysis where possible.

### Neuroimaging review

Brain MRI scans were reviewed locally but also by a neuroradiologist with significant experience in adult onset leukodystrophies (MA). Imaging features were documented using a structured template, assessing the presence, distribution, and progression of white matter abnormalities, as well as other relevant findings such as brain atrophy or diffusion restriction.

### Systematic literature review

To contextualise our findings, we performed a comprehensive review of the published literature on *AARS2-related* leukoencephalopathy. We searched PubMed using the terms "*AARS2*" AND "leukodystrophy" OR "leukoencephalopathy" from inception to 05/01/2026. Publications were included if they reported clinical cases with confirmed biallelic AARS2 variants. Demographical, clinical, radiological, and genetic data were extracted from each report. Duplicate cases described previously or insufficiently described reports were excluded, as were reports not in the English language.

## Results

### Cohort description

We identified 15 individuals with *AARS2*-related leukoencephalopathy (10 females and 5 males)—see Tables 1, 2. The mean age at onset was 28.5 years (range 0–46, SD 18.6). Most patients were compound heterozygotes (13/15), and two were homozygous. The cohort included two sibling pairs (individuals 5 and 6, and 9 and 10); the remaining eleven cases were unrelated.

**Table 1:** Demographics, clinical and radiological features of our 15-person cohort

Demography			Symptoms									Progression – Age of:			Imaging abnormality			
Pt Number	Gender	Age of Onset	Presenting complaint	Psychiatric	Cognitive	Pyramidal	Extrapyramidal motor feature	Gait disorder	Sphincter Disturbance	Seizure	Ovarian Failure	Wheelchair dependent	Incontinent	Dependent for ADLs	Diffuse white matter change	Diffusion restriction	Corpus callosum atrophy	Rarefied PV appearance on FLAIR
1	M	38	Behaviour	+	+	+	−	+	+	−	NA	40	39	40	+	+	+	NA
2	F	46	Cognitive	+	+	+	−	+	+	−	+	NA	47	NA	+	+	+	−
3	M	44	Personality	+	+	+	+	+	+	−	NA	NA	45	45	+	−	+	−
4	F	40	Global	+	+	+	+	+	+	−	NA	42	42	41	+	+	+	+
5	F	42	Psychosis	+	+	+	−	+	+	−	+	NA	42	42	+	−	+	+
6	M	42	Global	−	+	+	+	+	+	−	NA	44	42	42	+	−	+	+
7	M	35	Behaviour	+	+	+	+	+	+	+	NA	39	37	NA	+	−	+	NA
8	F	30	Aphasia	+	+	+	−	+	+	−	+	34	34	34	+	NA	+	NA
9	F	1	Developmental	+	+	−	+	+	+	+	+	27	25	25	+	+	+	NA
10	F	4	Developmental	+	+	+	+	+	−	−	+	NA	NA	NA	+	NA	−	NA
11	F	33	Behaviour	+	+	+	+	+	+	−	+	NA	34	33	+	NA	+	+
12	M	28	Psychosis	+	+	+	+	+	+	+	NA	31	30	32	+	−	+	+
13	F	0	Developmental	−	+	+	+	+	+	+	−	NA	NA	NA	+	+	+	+
14	F	0	Developmental	+	+	+	−	+	+	−	+	4	0	0	+	+	+	−
15	F	44	Behaviour	+	+	−	+	+	+	−	NR	44	45	44	+	−	+	+

**Table 2:** Genetic characterisation of our 15-person cohort

Pt Number	Zygosity	Allele 1					Allele 2					Reference
		Variant	**Type**	ACMG	Domain	gnomAD	Variant	Type	ACMG	Domain	gnomAD	
**1**	Heterozygous	c.595C > T; p.Arg199Cys ‡	Missense	Pathogenic	Catalytic	0.00007	g.(intron4)_(intron7)del (~ 4.1 kb) ‡	CNV	Pathogenic	NA	NA	[14]
**2**	Heterozygous	c.595C > T; p.Arg199Cys ‡	Missense	Pathogenic	Catalytic	0.00007	**c.1528A > G, p.Thr510Ala**	Missense	Likely path	Editing	NA	[2, 3, 5, 7, 13, 14, 30, 31]
**3**	Homozygous	c.650C > T; p.Pro217Leu ‡	Missense	Likely path	Catalytic	NA	c.650C > T; p.Pro217Leu ‡	Missense	Likely path	Catalytic	NA	[12, 24]
**4**	Heterozygous	c.892_894del; p.Gln298del ‡	In-frame deletion	Likely path	Catalytic	NA	c.1966del; p.His656llefs*11	Frameshift	Pathogenic	C-Terminal	NA	NA
**5**	Heterozygous	c.1774C > T; p.Arg592Trp †	Missense	Pathogenic	Editing	0.00020	c.1213G > A; p.Glu405Lys ‡	Missense	Likely path	Editing	0.00001	[5, 39]
**6**	Heterozygous	c.1774C > T; p.Arg592Trp †	Missense	Pathogenic	Editing	0.00020	c.1213G > A; p.Glu405Lys ‡	Missense	Likely path	Editing	0.00001	[5, 39]
**7**	Heterozygous	c.1082C > T; p.Ser361Phe †	Missense	Pathogenic	Catalytic	NA	**c.959C > T; p.Ala320Val**	Missense	Likely path	Catalytic	0.00000	[40]
**8**	Heterozygous	c.1082C > T; p.Ser361Phe †	Missense	Pathogenic	Catalytic	NA	c.2255 + 1G > A ‡	Splice	Pathogenic	NA	0.00001	[6, 40]
**9**	Heterozygous	c.149 T > G; p.Phe50Cys ‡	Missense	Pathogenic	N-Terminal	0.00000	c.964C > T; p.Arg322Cys †	Missense	Pathogenic	Catalytic	0.00001	[40]
**10**	Heterozygous	c.149 T > G; p.Phe50Cys ‡	Missense	Pathogenic	N-Terminal	0.00000	c.964C > T; p.Arg322Cys †	Missense	Pathogenic	Catalytic	0.00001	[40]
**11**	Heterozygous	c.595C > T; p.Arg199Cys ‡	Missense	Pathogenic	Catalytic	0.00007	**c.2780G > A; p.Cys927Tyr**	Missense	Likely path	C-Terminal	NA	[2, 3, 5, 7, 13, 14, 30, 31]
**12**	Heterozygous	c.1082C > T; p.Ser361Phe †	Missense	Pathogenic	Catalytic	NA	c.1329 T > A; p.Cys443*	Nonsense	Pathogenic	Editing	NA	[40]
**13**	Heterozygous	c.2699dup; p.Arg901Thrfs*5	Frameshift	Pathogenic	C-Terminal	NA	**c.190G > A; p.Val64Met**	Missense	Likely path	Catalytic	NA	NA
**14**	Heterozygous	c.790G > A; p.Val264Met †	Missense	Likely path	Catalytic	NA	c.2487 + 1G > C †	Splice	Pathogenic	NA	NA	[40]
**15**	Homozygous	c.595C > T; p.Arg199Cys ‡	Missense	Pathogenic	Catalytic	0.00007	c.595C > T; p.Arg199Cys ‡	Missense	Pathogenic	Catalytic	0.00007	[2, 3, 5, 7, 13, 14, 30, 31]

Variants are described using HGVS nomenclature based on transcript NM_020745.4. ACMG classification is based on ClinVar submissions where available. Protein domain assignment is derived from UniProt structural annotation of the canonical AARS2 protein (UniProt Q5JTZ9). Allele frequencies represent the global minor allele frequency in the Genome Aggregation Database (gnomAD). Novel variants identified in this cohort are indicated in bold. Variants previously associated only with cardiomyopathy in published literature and newly associated with leukodystrophy in this study are indicated with †—note c.1774C > T does have ClinVar associations for leukodystrophy. Variants previously published as associated with leukodystrophy are indicated with ‡—note c.2255 + 1G > A does have a ClinVar association for cardiomyopathy. References are provided for previously reported mutations

### Genetic findings

Across the cohort, we identified 19 unique *AARS2* variants amongst 15 patients, of which seven were novel. Two patients harboured biallelic novel variants (patients 4 and 13). Four patients carried one novel and one previously reported variant (patients 2, 7, 11, and 12). The remaining nine patients carried biallelic variants previously reported in the literature (patients 1, 3, 5, 6, 8, 9, 10, 14 and 15), including two individuals with a homozygous previously described variant (patients 3 and 15) [14, 24].

The most frequently observed previously reported variant was c.595C > T; p.Arg199Cys, present in four unrelated individuals (patients 1, 2, 11, and 15).

Four variants previously published only in association with cardiomyopathy—*c.1774C* > *T; p.Arg592Trp*, *c.1082C* > *T; p.Ser361Phe*, *c.964C* > *T; p.Arg322Cys*, and *c.790G* > *A; p.Val264Met*—were identified in this cohort and are reported here for the first time in association with a leukodystrophy phenotype. Notably, *c.1082C* > *T; p.Ser361Phe* was observed in three unrelated individuals (patients 7, 8, and 12).

### Clinical features

All patients exhibited progressive neurological symptoms consistent with *AARS2*-related leukoencephalopathy. Initial presentations commonly included psychiatric or behavioural change or cognitive decline. Paediatric-onset cases (4 individuals) showed global developmental impairment.

Where follow-up data were available, disease progression was typically rapid. Excluding one outlier patient who remained clinically stable for nearly 25 years, the mean time from symptom onset to wheelchair dependence was 2.6 years (median 2.5 years, range 0–4 years; n = 8). The mean time to urinary incontinence was 1.25 years (median 1 year, range 0–4 years; n = 12), whilst the mean time to loss of independence in activities of daily living was 1.2 years (median 0.5 years, range 0–4 years; *n* = 10).

### Neuroimaging findings

All patients in our cohort demonstrated confluent, asymmetric abnormal signal in the cerebral white matter, predominantly affecting the frontoparietal regions and sparing the subcortical u-fibres.

**Fig. 1:**
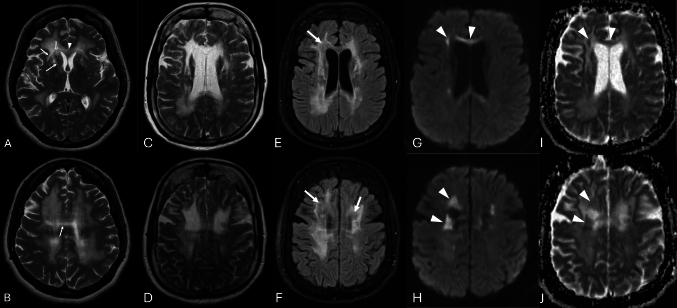
**A**, **B** T2-w axial images show confluent, asymmetric abnormal signal affecting the frontal and parietal white matter in Case 12. **A** Abnormal signal involves the anterior limb of the internal capsule and anterior parts of the external capsule (closed arrows), the anterior aspect of the caudate head (open arrow) and genu of the corpus callosum (arrowhead). The splenium of the corpus callosum and posterior limb of the internal capsule are spared. **B** Horizontal bands of severely affected white matter cross between the cerebral hemispheres via the corpus callosum. **C**, **D**, **E**, **F**, **G**, **H**, **I**, **J**) is Case 7. **C**, **D** T2-w axial images of asymmetric signal abnormalities present within the frontoparietal white matter and corpus callosum with patchy regions of white matter rarefaction (E, F) and foci of restricted diffusion, visible as areas of increased signal on the DWI trace images (**G**, **H**) corresponding to dark voxels on the ADC maps (**I**, **J**)

Several patients exhibited a frontal-predominant pattern involving the anterior limb of the internal capsule, anterior external capsule and the genu of the corpus callosum, with atrophy of the affected callosal segments. In one of these frontal-predominant cases, abnormal signal was also present within the heads of the caudate nuclei (Fig. 1). Of the 11 patients with available DWI, 6 had multifocal areas of restricted diffusion within the affected white matter (Fig. 1). Seven patients had regions of rarefied white matter visible as patchy foci of suppressed signal on the FLAIR sequences (Fig. 1). No patients harboured periventricular calcifications.

### Systematic literature review

We identified 56 genetically confirmed cases from 31 publications – see Supplementary Material 1**,** [2, 5–34]. The mean age of onset was 27.0 years (range 0.0–57.0, SD 13.5). Sex distribution was balanced (female n = 29, 51.8%). In total, 60 unique *AARS2* variants were described; 49 (87.5%) of patients were compound heterozygous and seven (12.5%) reported homozygous cases. The most frequently observed variant was *c.595C* > *T; p.Arg199Cys*, followed by *c.452 T* > *C; p.Met151Thr* and *c.179C* > *A; p.Pro60His*.

## Discussion

This study provides the most comprehensive synthesis of *AARS2*-related leukoencephalopathy to date, integrating 15 newly reported individuals with all 56 previously published cases (total = 71). Our findings refine the clinical, radiological, and genetic spectrum of the disorder and highlight both its defining features and its phenotypic breadth.

Across the combined cohort, the mean age at onset was 27.3 years, with near-equal sex distribution overall. *AARS2*-related leukoencephalopathy typically presents as a progressive adult-onset leukodystrophy but exhibits substantial heterogeneity. Psychiatric symptoms—including personality change, depression, and psychosis—were the presenting complaint in one-fifth of cases and ultimately occurred in 70% overall. Cognitive impairment was a similarly common feature, ultimately becoming nearly ubiquitous (86%), often becoming the dominant clinical issue (as seen in Case 3). Motor symptom progression was characterised by pyramidal involvement (82%), gait disturbance (80%), and extrapyramidal motor features (45%)—including tremor, dystonia, parkinsonism, chorea, and myoclonus.

Urinary incontinence occurred in over one-third of patients and frequently developed early in the disease course. Amongst patients with available longitudinal data, the mean time from symptom onset to urinary incontinence was 1.0 year (median 0 years, range 0–4 years; *n* = 15). Seizures were relatively uncommon (10%). Female reproductive involvement is nearly universal: 82% of women with available data exhibited ovarian failure (Case 2)—a number that may be higher with follow-up.

The mean time to wheelchair dependence was 2.1 years (median 1.5 years, range 0–8 years; *n* = 28). Mortality data were limited, but amongst seven reported deaths the median age at death was 28 years (mean 29.4 years, range 7–46 years), occurring a median of 4 years after symptom onset (mean 3.8 years, range 1–6.8 years).

Although adult-onset disease predominates, 16/71 (22.5%) individuals had childhood onset, including 12 with onset before age 12 (16.9%). Paediatric presentations were developmental, with seizures and learning difficulties; some progressed rapidly, whereas others remained stable for years before deteriorating in adolescence or adulthood (Case 11).

**Fig. 2:**
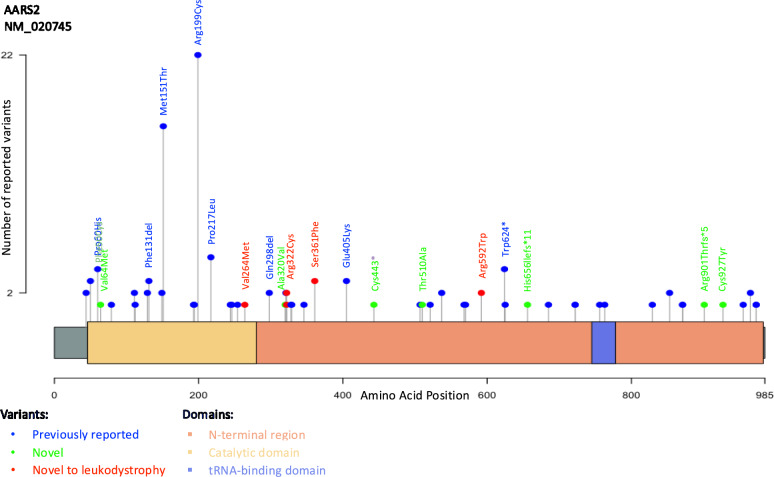
Lollipop plot showing the distribution of reported AARS2 protein-coding variants across the amino acid sequence. Each lollipop represents a unique variant positioned according to its predicted protein change (p.), with lollipop height indicating the number of times the variant has been reported. Novel variants are shown in green, whilst previously reported variants are shown in blue. Mutations previously only associated with cardiomyopathy and for the first time here associated with leukodystrophy are shown in red. Protein change annotations are shown for all novel variants, whereas only more common (> 2 reported cases) previously reported variants are labelled for clarity. Only variants with an unambiguous protein-level annotation were included; variants reported solely at the cDNA level (e.g. splice-site variants without an assigned protein consequence) or as multi-exonic deletions without defined protein breakpoints are not shown. The plot was generated in R using the maftools package

MR imaging demonstrates distinctive radiological patterns that can be highly suggestive of the diagnosis and are useful to recognise. Patients typically develop patchy then confluent, asymmetric abnormal signal in the frontoparietal white matter that begins close to the ventricular margins and extends towards the periphery, sparing the subcortical u-fibres. Frontal-predominant, parietal-predominant and frontoparietal patterns can be observed. When frontal white matter is extensively involved, the anterior limbs of the internal capsules and anterior aspects of the external capsules are often affected, sometimes extending into the heads of the adjacent caudate nuclei. Callosal involvement reflects the distribution of abnormalities in the cerebral white matter, and the affected segments are usually atrophic. Sharply demarcated bands of spared white matter may be seen passing from one hemisphere to the other via preserved segments of the corpus callosum (Fig. 1). Cerebral atrophy can occur in the affected lobes, principally seen as enlargement of the central cerebrospinal fluid (CSF) spaces without widening of the peripheral sulci (Fig. 2). Patchy white matter rarefaction and multifocal areas of restricted diffusion are often but not universally present.

Even when clinical and radiological features are strongly suggestive, a structured diagnostic approach remains essential. Modern evaluation of suspected adult-onset leukodystrophy begins with the exclusion of acquired causes, targeted biochemical assays of metabolic disease, and early involvement of specialist leukodystrophy services [35, 36] In the United Kingdom, this approach is exemplified by the Inherited White Matter Disorders Diagnostic and Management Service, a nationally commissioned service that coordinates specialist assessment and investigation of suspected inherited white matter disease via regular, virtual multi-disciplinary team meetings and in-person clinics [37].

Following this pathway, the combination of clinical features and the characteristic radiological pattern described above is likely to prompt early consideration of a genetic leukodystrophy. Within this context, two principal differential diagnoses arise beyond *AARS2*-related leukoencephalopathy.

*CSF1R-*RD is an important differential given the typical adult onset, striking pathological overlap, and shared radiological features including frontoparietal-predominant white matter involvement, corpus callosal abnormalities, pyramidal tract involvement, and diffusion restriction. *CSF1R*-RD however follows an autosomal dominant inheritance pattern, with an older age of onset and no association with ovarian failure. Further, DWI abnormalities in *AARS2*-related leukoencephalopathy tend to be larger than the punctate foci typical of *CSF1R*-RD and exhibit more marked reductions in ADC. The periventricular calcifications that can be seen on CT imaging of *CSF1R*-RD are also not present in cases of *AARS2*-related leukoencephalopathy.

Vanishing white matter disease (VWM; MIM # 603,896) is another important differential. VWM is an autosomal recessive disorder caused by biallelic pathogenic variants in one of five genes encoding the eukaryotic translation initiation factor eIF2B. It too can present with both early and late onset leukodystrophy and may be associated with ovarian failure in female patients. Radiologically, VWM is also characterised by progressive white matter rarefaction with areas of CSF-like signal intensity. Several clinical and radiological features can help differentiate *AARS2*-related leukoencephalopathy and VWM. VWM most commonly presents in childhood, with adult-onset disease being rare, and is typically marked by episodic or stepwise deterioration triggered by physiological stressors. On MRI, VWM lacks a consistent lobar predilection and characteristically spares the corpus callosum and long descending tracts. In addition, restricted diffusion is not a feature of VWM.

Targeted leukodystrophy gene panels via whole-exome or whole-genome sequencing now form the cornerstone of initial genetic testing and can reliably identify pathogenic variants in *AARS2* (as well as alternative diagnoses including *CSF1R*-RD and VWM). Standard sequencing pipelines may however miss pathogenic changes such as deep intronic, structural, or splicing-altering variants. Case 1 illustrates the diagnostic value of integrating RNA studies and whole-genome sequencing in such situations. When clinical suspicion remains high despite negative initial testing, further evaluation—including reanalysis of sequencing data, long-read sequencing, or functional RNA assays—should be considered.

Across all 71 individuals, we identified 72 unique *AARS2* variants, including seven variants that were not previously reported and five variants previously reported only in cardiomyopathy that are here associated with a leukodystrophy phenotype. Missense variants were the predominant class, consistent with a mechanism of partial loss of mt-AlaRS function. Truncating, frameshift, and splice-site variants were less frequent and typically occurred in trans with a missense allele. To visualise the distribution of *AARS2* variants across the protein, we mapped all reported variants with an unambiguous protein-level annotation onto the amino acid sequence (Fig. 2).

At the patient level, p.Arg199Cys and p.Met151Thr were the most frequent *AARS2* variants, affecting 28.2% and 18.3% of patients, respectively (corresponding to 15.5% and 11.3% of mutant alleles). p.Pro60His and p.Trp624* were identified in 5.6% and 4.2% of cases, respectively.

Some variants appear in both paediatric and adult-onset disease, whereas others, such as *p.Phe50Cys*, have thus far only been reported in childhood-onset presentations. Definitive genotype–phenotype correlations remain uncertain.

*AARS2* was initially implicated in fatal infantile-onset cardiomyopathy, a phenotype historically considered distinct from *AARS2*-related leukoencephalopathy. Early reports and subsequent reviews highlighted a strong association between cardiomyopathy and the recurrent editing-domain variant p.Arg592Trp, typically occurring either in homozygosity or in trans with a loss-of-function allele. This recurrent founder variant was originally described in multiple individuals with severe infantile hypertrophic cardiomyopathy and became regarded as a canonical cardiomyopathy-associated allele [38].

This apparent genotype–phenotype separation may not be absolute, however. Though unpublished, two recent ClinVar submissions have associated this variant with *AARS2*-related leukodystrophy (SCV005368011 and SCV006551412) [39]. Consistent with this observation, we identified the same variant in two affected siblings with leukodystrophy (patients 5 and 6) in our cohort. In addition, four other variants previously associated with infantile mitochondrial cardiomyopathy in ClinVar were identified in individuals with leukodystrophy in our cohort: *p.Ser361Phe* in three unrelated individuals (patients 7, 8 and 12), *p.Arg322Cys* in two affected siblings (patients 9 and 10), and both *p.Val264Met* and *c.2487* + *1G* > *C* in patient 14 [40]. To our knowledge, these four variants have not previously been reported in individuals with leukodystrophy in the published literature and are not currently associated with leukodystrophy in ClinVar. Together at least five alleles previously associated only with infantile cardiomyopathy can also seemingly manifest with a leukodystrophy phenotype.

Whilst previously reported *AARS2* leukodystrophy variants remain largely confined to neurological disease in the literature, our cohort expands the phenotypic spectrum of several cardiomyopathy-associated alleles and suggests that the proposed genotype–phenotype separation between cardiac and neurological disease is more porous than previously recognised.

This study has several limitations. The retrospective design and variable availability of imaging and longitudinal data limited uniform phenotyping. The literature review was constrained by inconsistent reporting across publications. Genetic diagnoses were established using heterogeneous platforms, and functional validation of novel variants was not universally feasible. In addition, estimates of disease progression, including time to wheelchair dependence, urinary incontinence, and death, were derived from relatively small numbers of patients with available longitudinal follow-up and should therefore be interpreted cautiously. Further collaborative studies with systematic prospective follow-up will be required to better define the natural history and prognostic trajectory of AARS2-related leukoencephalopathy. Despite these limitations, the combined dataset represents the most comprehensive characterisation of AARS2-related leukoencephalopathy to date.

## Illustrative case vignettes

### Case 3: A typical adult-onset neuropsychiatric presentation

A 45-year-old man presented with a six-month history of behavioural change and emerging double incontinence. Family members reported increasing apathy, disinhibition, and socially inappropriate behaviours. He became indifferent to personal hygiene and household responsibilities. Family history was notable for two uncles with young-onset dementia and gait disturbance. Brain MRI revealed extensive frontal-predominant white matter abnormalities, involvement of the anterior corpus callosum, and features suggestive of white matter rarefaction. Extensive metabolic, infectious, and autoimmune testing was unremarkable. Whole-genome sequencing via the R62 leukodystrophy panel revealed homozygous *AARS2* variants (*c.650C* > *T, p.Pro217Leu*), confirming the diagnosis.

### Case 2: A typical adult-onset presentation in a female patient

A 51-year-old woman presented with a five-year history of progressive behavioural and cognitive impairment. She had a long-standing history of primary ovarian failure, having experienced only one menstrual period in her life, and had been on lifelong hormone replacement therapy. Her family history was notable for tremor and psychiatric illness in her father and brother. From a high-functioning job, she became increasingly disoriented, developed apraxia and tremor, and required full support for ADLs. Brain MRI revealed confluent frontal white matter hyperintensity, marked involvement of the corpus callosum (particularly the genu), and patchy diffusion restriction. Whole-genome sequencing via the R62 leukodystrophy panel identified compound heterozygous variants in *AARS2* (*c.595C* > *T, p.Arg199Cys*; and *c.1528A* > *G, p.Thr510Ala).*

### Case 10: A typical child-onset neuro-developmental presentation

A 4-year-old girl presented with learning difficulties and behavioural changes following an initially normal early development. Later in childhood she would develop psychiatric symptoms (persecutory delusions, auditory hallucinations), as well as progressive gait dysfunction and speech impairment. She had growth hormone deficiency, delayed pubertal signs, premature ovarian failure and small ovaries on imaging. Neurological exam revealed global ataxia. MRI revealed deep white matter abnormalities and marked cerebellar atrophy. WES revealed *c.149 T* > *G; p.Phe50Cys* and *c.964C* > *T; p.Arg322Cys AARS2* variants—also found in her sister (patient 9). The patient remains stable, independent in all activities of daily living.

### Case 1: An example of a case where routine WGS failed to detect an AARS2 mutation.

A 38-year-old man presented with apathy, disinhibition, and cognitive decline, progressing to spastic tetraplegia and mutism by age 40. MRI showed characteristic features of *AARS2*-related leukoencephalopathy, including deep white matter diffusion dots and corpus callosum atrophy. Despite high clinical diagnostic suspicion, focussed exome and Sanger sequencing identified only one heterozygous pathogenic variant (*c.595C* > *T*). To investigate the missing second allele, RNA studies were performed on patient-derived fibroblasts which demonstrated monoallelic expression of the mutant transcript, suggesting the presence of a second, transcriptionally silent allele. Subsequent whole-genome sequencing identified a heterozygous ~ 4.1 kb intragenic deletion spanning intron 4 to intron 7 of *AARS2*, with an inserted 59 bp sequence. Deletion-specific PCR and breakpoint sequencing confirmed the structural variant. cDNA analysis was consistent with loss of expression from the deleted allele.

## Conclusion

This multicentre case series and literature review highlight *AARS2*-related leukoencephalopathy as a clinically and genetically heterogeneous leukodystrophy spanning infancy to late adulthood with characteristic neuroimaging features. Our findings also suggest that the previously proposed separation between cardiomyopathy- and leukodystrophy-associated *AARS2* variants is not absolute, with several variants historically linked to infantile cardiomyopathy observed in individuals with leukodystrophy in our cohort. Early recognition and appropriate genomic testing are therefore essential for timely diagnosis. Greater awareness of the breadth of clinical and genetic presentations will aid clinicians in differentiating *AARS2*-related disease from other adult-onset leukodystrophies and improve diagnostic accuracy.

## Supplementary Information

Below is the link to the electronic supplementary material.

Supplementary file1 (DOCX 177 KB)

## Data Availability

The datasets generated and/or analysed during the current study are not publicly available due to patient confidentiality and institutional restrictions but may be available from the corresponding author on reasonable request.
