# Supplementary material for: The clinical, radiological and genetic spectrum AARS2-related leukoencephalopathy: a case series of 15 patients and review of the literature
Source: J Neurol. 2026 Jun 26;273(7):426. doi: 10.1007/s00415-026-13964-1 (PMC13309504; doi:10.1007/s00415-026-13964-1)
Supplement: Supplementary file 1 — Supplementary file1 (DOCX 177 KB) [file 415_2026_13964_MOESM1_ESM.docx]

**Supplementary Material: Literature Review**

| **Demography** | | | | **Genetics** | | **Symptoms** | | | | | | | | | **Progression – Age of:** | | | **Imaging Abnormality** | | | | **Reference** |
| --- | --- | --- | --- | --- | --- | --- | --- | --- | --- | --- | --- | --- | --- | --- | --- | --- | --- | --- | --- | --- | --- | --- |
| **Pt Number** | **Gender** | | **Age of Onset** | **Variant (Allele 1)** | **Variant (Allele 2)** | **Presenting complaint** | **Psychiatric** | **Cognitive** | **Pyramidal** | **Extrapyramidal motor feature** | **Gait disorder** | **Sphincter Disturbance** | **Seizure** | **Ovarian Failure** | **Wheelchair dependent** | **Incontinent** | **Death** | **Diffuse white matter change** | **Diffusion restriction** | **Corpus callosum atrophy** | **Rarefied PV appearance on FLAIR** |  |
| 1 | | F | 28.0 | *c.1609C>T; p.Gln537SeM* | *c.595C>T; p.Arg199Cys* | Depression | + | + | + | - | + | NR | + | + | 36 | NR | NR | + | NR | + | + | ^1^ |
| 2 | | F | 23.0 | *c.230C>T; p.Gly965Arg* | *c.595C>T; p.Arg199Cys* | Tremor | + | + | + | - | + | NR | - | + | NR | NR | 28 | + | + | + | + |  |
| 3 | | F | 40.0 | *c.595C>T; p.Arg199Cys* | *c.390_392del; p.Phe131del* | Cognitive | + | + | + | - | + | NR | - | + | NR | NR | 46 | + | NR | + | + |  |
| 4 | | F | 20.0 | *c.595C>T; p.Arg199Cys* | *c.2611dup; p.Thr871fs* | Balance | + | + | + | - | + | NR | - | + | 23 | NR | NR | + | NR | + | + |  |
| 5 | | F | 3.0 | *c.149T>G; p.Phe50Cys* | *c.1561C>T; p.Arg521Ter* | Balance | + | + | + | - | + | NR | - | + | NR | NR | NR | - | - | - | + |  |
| 6 | | M | 7.0 | *c.2893G>A; p.Gly965Arg* | *c.1213G>A; p.Glu405Lys* | Developmental | - | + | + | + | + | NR | - | NA | NR | NR | NR | + | - | + | + |  |
| 7 | | F | 31.0 | *c.1145C>A* | *c.2255+1G>A* | Cognitive | + | + | + | + | + | + | - | + | 38 | 31 | NR | + | + | + | + | ^2^ |
| 8 | | M | 18.0 | *c.578T>G; p.Leu193** | *c.595C>T; p.Arg199Cys* | Behaviour | + | + | + | + | + | NR | - | NA | 24 | NR | NR | + | NR | + | NR | ^3^ |
| 9 | | F | 26.0 | *c.1041-1G>A* | *c.595C>T; p.Arg199Cys* | Cognitive | + | + | + | NR | + | NR | NR | + | NR | NR | NR | + | NR | NR | NR | ^4^ |
| 10 | | M | 37.0 | *c.1188G>A* | *c.1709delG* | Behaviour | + | + | NR | + | + | NR | NR | NA | 38 | NR | NR | + | + | + | + |  |
| 11 | | M | 25.0 | *c.1188G>A* | *c.1709delG* | Motor | - | + | NR | NR | + | NR | NR | NA | 26 | NR | 26 | + | NR | NR | NR |  |
| 12 | | M | 15.0 | *c.892_894del* | *c.2234_2235* | Behaviour | + | + | + | + | + | NR | NR | NA | 16 | NR | 18 | + | + | + | + |  |
| 13 | | M | 44.0 | *c.595C>T; p.Arg199Cys* | *c.595C>T; p.Arg199Cys* | Dystonia | + | + | + | + | NR | NR | NR | NA | 45 | NR | 45 | + | NR | NR | NR |  |
| 14 | | F | 33.0 | *c.963C>A; p.Tyr321** | *c.452T>C; p.Met151Thr* | Gait | + | + | + | + | + | NR | - | + | 35 | NR | NR | + | + | + | + | ^5^ |
| 15 | | M | 35.0 | *c.963C>A; p.Tyr321** | *c.452T>C; p.Met151Thr* | Cognitive | + | + | + | + | + | + | - | NA | 37 | 37 | NR | + | + | + | + |  |
| 16 | | M | 1.5 | *c.1519G>C; p.Val507Leu* | *c.2165G>A; p.Arg722Gln* | Vision | NR | NR | + | NR | NR | NR | NR | NA | NR | NR | NR | + | + | + | NR | ^6^ |
| 17 | | F | 27.0 | *C.1871G>A; p.Trp624** | *c.452T>C; p.Met151Thr* | Gait | + | + | + | NR | + | NR | NR | - | NR | NR | NR | + | + | NR | + | ^7^ |
| 18 | | M | 17.0 | *c.2265dupA; p.Arg756fs* | *c.650C>T; p.Pro217Leu* | Tremor | + | + | + | + | + | - | NR | NA | 22 | NR | NR | + | + | + | + | ^8^ |
| 19 | | F | 44.0 | *c.595C>T; p.Arg199Cys* | *c.390_392del; p.Phe131del* | Gait | - | + | NR | NR |  | NR | NR | + | NR | NR | NR | + | + | + | + | ^9^ |
| 20 | | F | 32.0 | *c.595C>T; p.Arg199Cys* | *c.236T>A; p.Met79Lys* | Cognitive | + | + | + | NR | + | NR | NR | + | 36 | NR | 36 | + | NR | NR | + |  |
| 21 | | F | 23.0 | *c.595C>T; p.Arg199Cys* | *c.2611_2612insA; p.Thr871Asnfs* | Gait | - | - | + | NR | + | NR | NR | + | NR | NR | NR | + | - | + | + |  |
| 22 | | F | 34.0 | *c.1691T>C* | *c.179C>A; p.Pro60His* | Motor | + | + | + | - | + | + | NR | - | NR | NR | NR | + | + | + | + | ^10^ |
| 23 | | M | 29.0 | *c.179C>A; p.Pro60His* | *c.1703_1704del; p.Gln568fs* | Motor | - | - | + | + | + | - | - | NA | NR | NR | NR | + | + | + | + | ^11^ |
| 24 | | M | 42.0 | *c.595C>T; p.Arg199Cys* | *c.2557C>T; p.Arg853Trp* | Tremor | + | + | + | - | + | + | + | NA | 46 | 42 | NR | + | + | + | + | ^12^ |
| 25 | | M | 34.0 | *c.595C>T; p.Arg199Cys* | *c.2598+1G>T* | Tremor | + | + | + | + | + | + | - | NA | NR | NR | NR | + | NR | + | - |  |
| 26 | | F | 57.0 | *c.595C>T; p.Arg199Cys* | *Deletion of exons 5-7* | Tremor | + | + | + | + | + | NR | - | NR | NR | NR | NR | + | NR | + | - |  |
| 27 | | M | 44.0 | *c.452T>C; p.Met151Thr* | *c.452T>C; p.Met151Thr* | Gait | + | + | + | - | + | NR | - | NA | NR | NR | NR | + | + | + | + | ^13^ |
| 28 | | M | 22.0 | *c.130G>C; p.Ala44Pro* | *c.130G>C; p.Ala44Pro* | Motor | + | + | + | - | + | NR | - | NA | NR | NR | NR | + | - | + | NR | ^14^ |
| 29 | | M | 20.0 | *c.965G>A; p.Arg322His* | *c.334G>C; p.Gly112Arg* | Gait | + | + | + | - | + | NR | - | NA | NR | NR | NR | + | + | + | + | ^15^ |
| 30 | | M | 28.0 | *c.452T>C; p.Met151Thr* | *c.737T>A; p.Met246Lys* | Gait | - | - | + | NR | NR | NR | NR | NA | NR | NR | NR | + | NR | + | + | ^16^ |
| 31 | | F | 26.0 | *c.390-392del; p.Phe131del_* | *c.984C>G; p.Ile328Met* | Dysarthria | - | - | + | - | + | - | - | - | NR | NR | NR | - | - | - | - | ^17^ |
| 32 | | F | 22.0 | *C.1871G>A; p.Trp624** | *c.452T>C; p.Met151Thr* | Tremor | - | + | + | + | + | NR | - | + | NR | NR | NR | NR | NR | NR | NR | ^18^ |
| 33 | | M | 0.3 | *C.1871G>A; p.Trp624** | *c.802A>G; p.Gln685fs* | Dyskinesia | + | + | + | + | + | + | - | NA | NR | NR | NR | + | + | + | + |  |
| 34 | | M | 38.0 | *c.452T>C; p.Met151Thr* | *c.452T>C; p.Met151Thr* | Behaviour | + | + | + | - | + | NR | - | NA | NR | NR | NR | + | + | + | + |  |
| 35 | | M | 12.0 | *C.1871G>A; p.Trp624** | *c.452T>C; p.Met151Thr* | Motor | + | + | + | - | + | NR | - | NA | NR | NR | NR | + | + | + | + |  |
| 36 | | F | 0.2 | *c.1150-4C>G* | *c.1150-4C>G* | Developmental | - | + | + | + | + | + | + | NR | NR | NR | 7 | + | NR | + | NR | ^19^ |
| 37 | | F | 9.0 | *c.2682+5G>A** | *c.331G>C; p.Ala111Pro* | NR | NR | NR | NR | NR | NR | NR | NR | NR | NR | NR | NR | NR | NR | NR | NR | ^20^ |
| 38 | | M | 8.0 | *c.2682+5G>A** | *c.331G>C; p.Ala111Pro* | NR | NR | NR | NR | NR | NR | NR | NR | NR | NR | NR | NR | NR | NR | NR | NR |  |
| 39 | | M | 0.7 | *c.2164C>T; p.Arg722Trp* | *c.761G>A; p.Gly254Ala* | NR | NR | NR | NR | NR | NR | NR | NR | NR | NR | NR | NR | NR | NR | NR | NR |  |
| 40 | | F | 38.0 | *c.385A_>_C; p.Thr129Pro* | *c.446G_>_A; p.Cys149Tyr* | Tremor | - | - | + | + | + | - | - | + | NR | NR | NR | - | NR | NR | NR | ^21^ |
| 41 | | F | 32.0 | *c.385A_>_C; p.Thr129Pro* | *c.446G_>_A; p.Cys149Tyr* | Tremor | - | - | + | + | + | - | - | + | NR | NR | NR | - | NR | NR | NR |  |
| 42 | | M | 27.0 | *c.650C>T; p.Pro217Leu* | *c.650C>T; p.Pro217Leu* | Cognitive | + | + | + | - | + | + | - | NA | 28 | 27 | NR | + | + | + | + | ^22^ |
| 43 | | M | 13.0 | *c.647dupG* | *c.595C>T; p.Arg199Cys* | Psychosis | + | + | + | + | + | - | - | NA | NR | NR | NR | + | NR | + | NR | ^23^ |
| 44 | | M | 21.0 | *c.452T>C; p.Met151Thr* | *c.2557C>T; p.Arg853Trp* | Gait | - | + | + | + | + | + | - | NA | 22 | 22 | NR | + | + | + | + | ^24^ |
| 45 | | F | 24.0 | *c.718C>T* | *c.1040+1G>A* | Motor | + | + | + | + | + | - | - | + | NR | NR | NR | + | + | + | + | ^25^ |
| 46 | | M | 55.0 | *c.1874G>A; p.Arg625His* | *c.179C>A; p.Pro60His* | Gait | + | + | + | + | + | - | - | NA | NR | NR | NR | + | + | + | + | ^26^ |
| 47 | | M | 28.0 | *c.452T>C; p.Met151Thr* | *c.2146_2A>G* | Cognitive | + | + | + | - | + | + | - | NA | NR | NR | NR | + | + | + | + | ^27^ |
| 48 | | F | 41.0 | *c.2864G>A p.Trp955** | *c.1036C>A; p.Pro346Thr, p.Pro346Wfs*18* | Cognitive | + | + | + | - | + | - | - | + | 43 | NR | NR | + | + | + | NR | ^28^ |
| 49 | | F | 25.0 | *c.595C>T; p.Arg199Cys* | *c.1885A>G; p.Met829Val* | Tremor | + | + | + | + | + | - | - | NR | 27 | NR | NR | + | NR | + | NR | ^29^ |
| 50 | | M | 40.0 | *c.595C>T; p.Arg199Cys* | *c.730G > A; p.Val244Ile* | Headache | + | + | - | - | - | - | - | NA | NR | NR | NR | + | - | + | - | ^30^ |
| 51 | | F | 30.0 | *c.986G>A; p.Arg329His* | *c.581G>A; p.Gly194Glu* | Behaviour | + | + | - | - | NR | - | - | + | NR | NR | NR | + | + | + | + | ^31^ |
| 52 | | F | 36.0 | *c.452T>C; p.Met151Thr* | *c.452T>C; p.Met151Thr* | Cognitive | - | + | - | - | NR | + | - | - | NR | NR | NR | + | + | + | + |  |
| 53 | | F | 35.0 | *c.179C>A; p.Pro60His* | *c.1708G>C; p.Gly570Arg* | Cognitive | - | + | - | - | NR | - | - | - | NR | NR | NR | + | + | + | + |  |
| 54 | | F | 41.0 | *c.2917G>A; p.Ala973Thr* | *c.2918C>A; p.Ala973Asp* | Behaviour | + | + | + | - | NR | - | - | + | NR | NR | NR | + | - | - | - |  |
| 55 | | F | 29.0 | *c.2288T>C; p.Leu763Pro* | *c.452T>C; p.Met151Thr* | Cognitive | + | + | + | - | NR | + | - | + | NR | NR | NR | + | + | + | + |  |
| 56 | | F | 44.0 | *c.1609C>T; p.Gln537SeM* | *c.452T>C; p.Met151Thr* | Gait | - | + | - | - | NR | - | - | + | NR | NR | NR | + | + | + | + |  |

+ = present; − = absent; NR = not reported; NA = not applicable

**References**

1. Dallabona C, Diodato D, Kevelam SH, et al. Novel (ovario) leukodystrophy related to AARS2 mutations. Neurology ® 2014.

2. Hamatani M, Jingami N, Tsurusaki Y, et al. The first Japanese case of leukodystrophy with ovarian failure arising from novel compound heterozygous AARS2 mutations. J Hum Genet [online serial]. 2016;61:899–902. Accessed at: https://www.nature.com/articles/jhg201664.

3. Szpisjak L, Zsindely N, Engelhardt JI, Vecsei L, Kovacs GG, Klivenyi P. Novel AARS2 gene mutation producing leukodystrophy: A case report. J Hum Genet. Nature Publishing Group; 2017;62:329–333.

4. Lynch DS, Zhang WJ, Lakshmanan R, et al. Analysis of mutations in AARS2 in a series of CSF1R-negative patients with adult-onset leukoencephalopathy with axonal spheroids and pigmented glia. JAMA Neurol. American Medical Association; 2016;73:1433–1439.

5. Lee JM, Yang HJ, Kwon JH, et al. Two Korean siblings with recently described ovarioleukodystrophy related to AARS2 mutations. Eur. J. Neurol. Blackwell Publishing Ltd; 2017. p. e21–e22.

6. Peragallo JH, Keller S, van der Knaap MS, Soares BP, Shankar SP. Retinopathy and optic atrophy: Expanding the phenotypic spectrum of pathogenic variants in the AARS2 gene. Ophthalmic Genet. Taylor and Francis Ltd; 2018;39:99–102.

7. Sun J, Quan C, Luo SS, Zhou L, Zhao CB. Leukodystrophy without ovarian failure caused by compound heterozygous alanyl-tRNA synthetase 2 mutations. Chin. Med. J. (Engl). Wolters Kluwer Medknow Publications; 2017. p. 3021–3022.

8. Dong Q, Long L, Chang YY, Lin YJ, Liu M, Lu ZQ. An adolescence-onset male leukoencephalopathy with remarkable cerebellar atrophy and novel compound heterozygous AARS2 gene mutations: A case report. J Hum Genet. Nature Publishing Group; 2018;63:841–846.

9. Taglia I, Di Donato I, Bianchi S, et al. AARS2-related ovarioleukodystrophy: Clinical and neuroimaging features of three new cases. Acta Neurol Scand. Blackwell Publishing Ltd; 2018;138:278–283.

10. Wang D, Yu M, Zhang W, Wang Z, Yuan Y. AARS2 compound heterozygous variants in a case of adult-onset leukoencephalopathy with axonal spheroids and pigmented glia. J Neuropathol Exp Neurol. Oxford University Press; 2018;77:997–1000.

11. Tang Y, Qin Q, Xing Y, Guo D, Di L, Jia J. AARS2 leukoencephalopathy: A new variant of mitochondrial encephalomyopathy. Mol Genet Genomic Med. Wiley-Blackwell; 2019;7.

12. Srivastava S, Butala A, Mahida S, et al. Expansion of the clinical spectrum associated with AARS2-related disorders. Am J Med Genet A. Wiley-Liss Inc.; 2019;179:1556–1564.

13. Wang JY, Chen SF, Zhang HQ, Wang MY, Zhu JH, Zhang X. A homozygous mutation of alanyl-transfer RNA synthetase 2 in a patient of adult-onset leukodystrophy: A case report and literature review. Brain Behav. John Wiley and Sons Ltd; 2019;9.

14. Uzun GA. Adult-onset leukodystrophy with homozygous AARS2 mutation located in the aminoacylation domain. Neurol. India Wolters Kluwer Medknow Publications; 2019. p. 871–872.

15. Song C, Peng L, Wang S, Liu Y. A novel compound heterozygous mutation in AARS2 gene (c.965 G > A, p.R322H; c.334 G > C, p.G112R) identified in a Chinese patient with leukodystrophy involved in brain and spinal cord. J Hum Genet. Nature Publishing Group; 2019;64:979–983.

16. Seo GH, Oh A, Kim EN, et al. Identification of extremely rare mitochondrial disorders by whole exome sequencing. J Hum Genet. Nature Publishing Group; 2019;64:1117–1125.

17. Kuo ME, Antonellis A, Shakkottai VG. Alanyl-tRNA Synthetase 2 (AARS2)-Related Ataxia Without Leukoencephalopathy. Cerebellum. Springer; 2020;19:154–160.

18. Wang X, Wang Q, Tang H, et al. Novel Alanyl-tRNA Synthetase 2 Pathogenic Variants in Leukodystrophies. Front Neurol. Frontiers Media S.A.; 2019;10.

19. Felhi R, Charif M, Sfaihi L, et al. Mutations in aARS genes revealed by targeted next-generation sequencing in patients with mitochondrial diseases. Mol Biol Rep. Springer; 2020;47:3779–3787.

20. Wu TH, Peng J, Zhang CL, et al. Mutations in aminoacyl-tRNA synthetase genes: An analysis of 10 cases. Chinese Journal of Contemporary Pediatrics. Xiangya Hospital of CSU; 2020;22:595–601.

21. De Michele G, Galatolo D, Lieto M, et al. New AARS2 Mutations in Two Siblings With Tremor, Downbeat Nystagmus, and Primary Amenorrhea: A Benign Phenotype Without Leukoencephalopathy. Mov Disord Clin Pract. Wiley-Blackwell; 2020;7:684–687.

22. Axelsen TM, Vammen TL, Bak M, Pourhadi N, Stenør CM, Grønborg S. Case report: ‘AARS2 leukodystrophy.’ Mol Genet Metab Rep. Elsevier Inc.; 2021;28.

23. Parra SP, Heckers SH, Wilcox WR, Mcknight CD, Jinnah HA. The emerging neurological spectrum of AARS2-associated disorders. Parkinsonism Relat. Disord. Elsevier Ltd; 2021. p. 50–54.

24. Zhang X, Li J, Zhang Y, Gao M, Peng T, Tian T. AARS2-Related Leukodystrophy: a Case Report and Literature Review. Cerebellum. Springer; 2023;22:59–69.

25. Fan Y, Han J, Yang Y, Chen T. Novel mitochondrial alanyl-tRNA synthetase 2 (AARS2) heterozygous mutations in a Chinese patient with adult-onset leukoencephalopathy. BMC Neurol. BioMed Central Ltd; 2022;22.

26. Chakraborty AP, Mukherjee A, Bhattacharyya A, Bhattacharyya D, Ray BK, Biswas A. Gait Apraxia with Exaggerated Upper Limb Movements as Presentation of AARS2 Related Leukoencephalopathy. Tremor and Other Hyperkinetic Movements. Center for Digital Research and Scholarship; 2022;12.

27. Li Y, Xu J, Xu Y, Li C, Wu Y, Liu Z. Clinical, genetic, and molecular characteristics in a central-southern Chinese cohort of genetic leukodystrophies. Ann Clin Transl Neurol. John Wiley and Sons Inc; 2023;10:1556–1568.

28. Kazakova E, Téllez-Martínez JA, Flores-Lagunes L, et al. Uterus infantilis: a novel phenotype associated with AARS2 new genetic variants. A case report. Front Neurol. Frontiers Media SA; 2023;14.

29. Green K, Maciver CL, Ebden S, Rees DA, Peall KJ. Pearls & Oy-sters: AARS2 Leukodystrophy - Tremor and Tribulations. Neurology. Lippincott Williams and Wilkins; 2024;102.

30. Fernandes J, Moura J, Tarrio J, et al. A novel disease-causing variant associated with a milder phenotype of AARS2-related leukodystrophy — A case report. Mol Genet Metab Rep. Elsevier Inc.; 2024;41.

31. Mao C, Qiu Y, Wang T, et al. Clinical Diagnosis and Differential Diagnosis Between CSF1R- and AARS2-Related Leukoencephalopathy. Journal of Molecular Neuroscience. Springer; 2025;75.
